# Supplementary material for: G Protein-Dependent Activation of the PKA-Erk1/2 Pathway by the Striatal Dopamine D1/D3 Receptor Heteromer Involves Beta-Arrestin and the Tyrosine Phosphatase Shp-2
Source: Biomolecules. 2023 Mar 3;13(3):473. doi: 10.3390/biom13030473 (PMC10046256; doi:10.3390/biom13030473)
Supplement: Supplementary file 1 [file biomolecules-13-00473-s001.zip › biomolecules-2181776-supplementary.pdf]

G protein-dependent activation of the PKA-Erk1/2 pathway by the striatal dopamine D1/D3 receptor heteromer involves beta-arrestin and the tyrosine phosphatase Shp-2.

Federica Bono[1]†, Zaira Tomasoni[1]†, Veronica Mutti[1], Giulia Sbrini[1], Rajesh Kumar[2], Francesca Longhena[1], Chiara Fiorentini[1]#* and Cristina Missale[1]#

Supplementary materials

S1. In Hek-D1R cells, D1R activation promotes Erk1/2 phosphorylation, but not Akt, through the cAMP/PKA pathway

Hek293 cells transiently expressing D1R (Hek-D1R) were treated with the D1R agonist SKF81297 (1 μM) for 5 min and analysed for activation of Erk1/2 and Akt pathways. Phosphorylation of Erk1/2 (p-Erk1/2) and Akt (p-Akt) were analysed on total protein preparations by using Western Blot (WB) analyses. In line with previous data [11-12, 36], we found that D1R activated Erk1/2 in a time-dependent manner with a peak of activation occurring at 5 minutes (min) and a rapid return to the basal level within 15 min of D1R stimulation (Figure S1A and B). By contrast, in this cell model, D1R stimulation did not promote Akt phosphorylation (Figure S1A and C). In addition, p-Erk1/2 induced by SKF81297 (1 μM, 5 min) was specifically abolished by the PKA inhibitor H89 (1 μM), added 30 min before agonist stimulation; by contrast, pre-incubation with LY294002 (10 μM), a specific inhibitor of the phosphoinositide 3 kinase (PI3-K), did not affect the capability of D1R to activate Erk1/2 (Figure S1D and E). Incubation of Hek-D1R cells with H89 (1 μM) and LY29002 (10 μM) did not change p-Erk1/2 basal levels (Figure S1F and G). These results indicate that in Hek293 cells D1R stimulation leads to Erk1/2 activation, but not Akt, in a PKA-dependent manner.

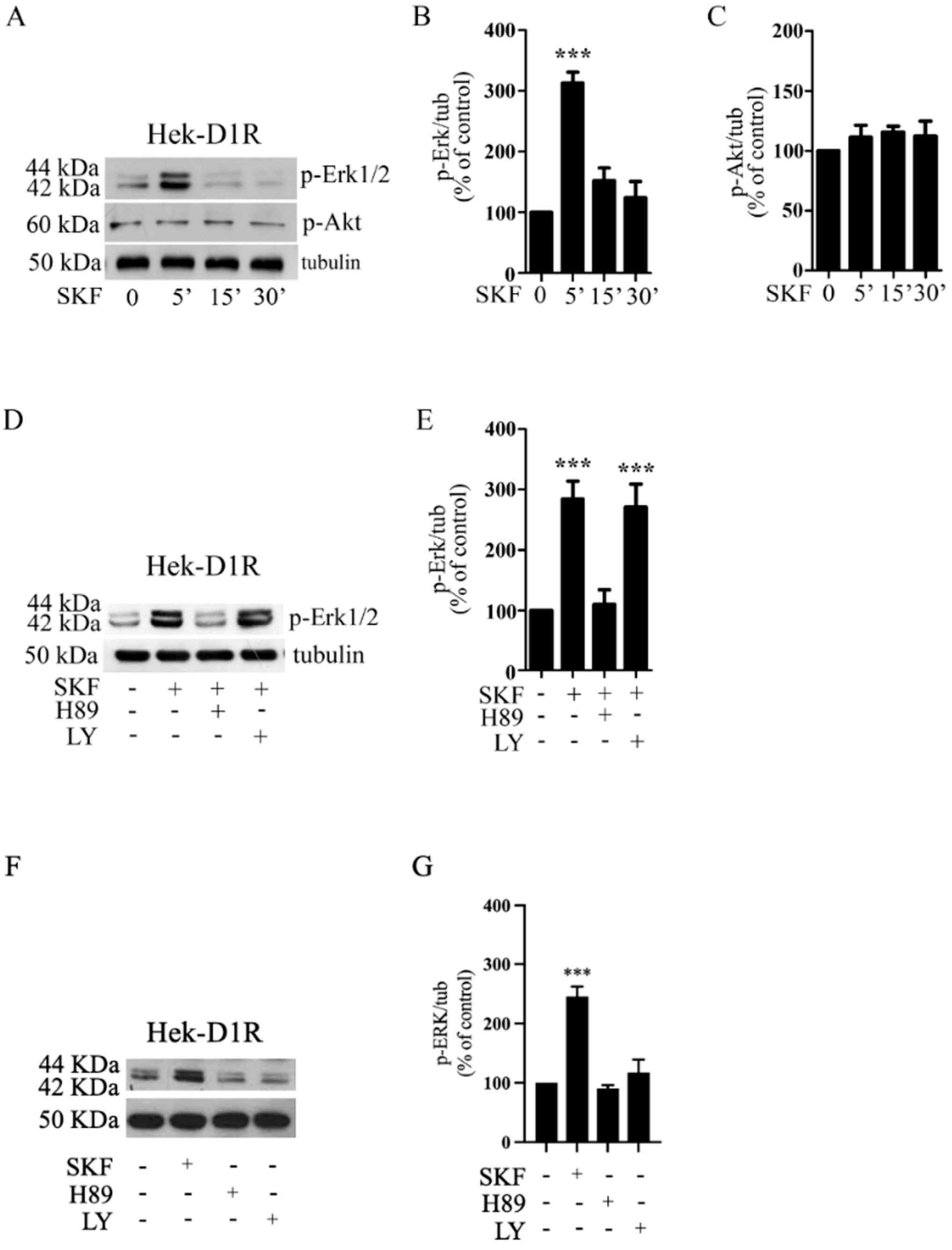

Figure S1. D1R activation of Erk1/2 in Hek-D1R expressing cells. (A) Hek293 cells transiently expressing the D1R (Hek-D1R) were treated with the D1R agonist SKF81297 (SKF, 1 μM) for 5-30 min and analyzed for Erk1/2 and Akt phosphorylation (p-Erk1/2 and p-Akt, respectively) by WB; a representative WB is shown. (B) Densitometric analysis of blots (n = 4) with specific p-Erk1/2 levels normalized to the corresponding tubulin levels. Bars represent the mean ±S.E.M. *** p<0.01 vs untreated cells. (C) Densitometric analysis of blots (n = 4) with specific p-Akt levels normalized to the corresponding tubulin levels. Bars represent the mean ±S.E.M. (D) Hek-D1R cells were treated with SKF (1 μM; 5 min) in the absence or in the presence of both H89 (1 μM) and LY294002 (LY; 10 μM) and analysed for p-Erk1/2 by WB; a representative WB is shown. (E) Densitometric analysis of blots (n = 4) with specific p-Erk1/2 levels normalized to the corresponding tubulin levels. Bars represent the mean ±S.E.M. *** p<0.01 vs untreated cells. (F) Hek-D1R cells were treated with SKF (1 μM; 5 min), H89 (1 μM, 30 min) or LY294002 (LY; 10 μM, 30 min) and analysed for p-Erk1/2 by WB; a representative WB is shown. (G) Densitometric analysis of blots (n = 4) with specific p-Erk1/2 levels normalized to the corresponding tubulin levels. Bars represent the mean ±S.E.M. *** p<0.01 vs untreated cells. Data were statistically analyzed by one-way ANOVA followed by post-hoc comparison with Bonferroni test.

S2. In Hek-D3R cells, D3R induces Erk1/2 and Akt phosphorylation in a Gi/o protein/PI3-K dependent pathway

D3R activation of both Erk1/2 and Akt was previously reported in different cell models [15, 37-41]; the mechanisms underlying D3R-mediated activation of these pathways were analysed in Hek293 transiently expressing D3R (Hek-D3R). Cells were treated with the D2R/D3R agonist quinpirole (1 μM) for different times; in addition, stimulation with quinpirole was performed in the presence or in the absence of H89 (1 μM) or LY294002 (1 μM), added 30 min prior to D3R stimulation. The role of Gi/o protein was also evaluated by using pertussis toxin (PTX) that, catalysing the ADP-rybosilation of the alpha subunit, prevents their interaction with the G-protein coupled receptor (GPCR). To this end, Hek-D3R cells were incubated overnight with PTX (100 ng/ml) following by quinpirole incubation (1 μM) for 5 min. A time-dependent activation of Erk1/2 was observed that reached a maximum at around 5 min of receptor stimulation and returned to basal levels after 30 min of receptor stimulation (Figure S2A and B). This effect was blocked by the PI3-K inhibitor, LY294002 (Figure S2D and E) as well as by PTX (Figure S2G and H). Otherwise, the PKA inhibitor, H89, did not modify D3R-induced Erk1/2 phosphorylation (Figure S2D and E). A more prolonged phosphorylation of Akt was also induced by D3R stimulation, with a pick occurring at 5 min of D3R activation (Figure S2A and C). Similarly, to that observed for phosphorylation of Erk1/2, D3R-induced p-Akt, measured following 5 min of D3R stimulation, was prevented by both LY294002 and PTX (Figure S2D, F, G and I). Incubation of Hek-D3R cells with LY29002 (10 μM) did not change p-Erk1/2 and p-Akt basal levels (Figure 2SJ, K and L). Therefore, in Hek-D3R cells, activation of the Erk1/2/Akt pathway by D3R occurs through a PTX-sensitive G protein mechanism and requires PI3-K.

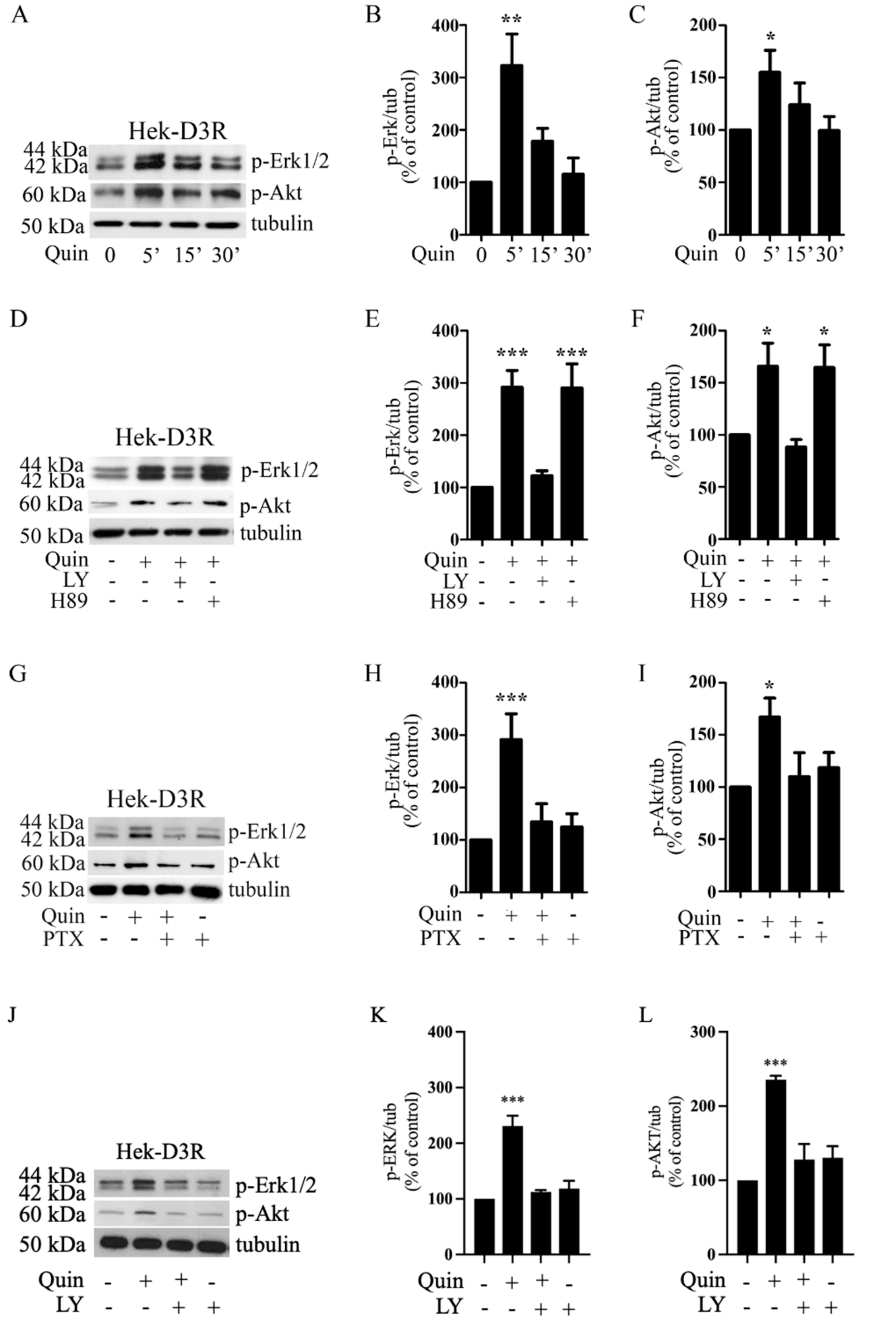

Figure S2. D3R activation of Erk1/2 and Akt in Hek-D3R expressing cells. (A) Hek cells transiently expressing the D3R (Hek-D3R) were treated with the D2R/D3R agonist quinpirole (Quin, 1 μM) for 5-30 min and analyzed for p-Erk1/2 and p-Akt by WB; a representative WB is shown. (B) Densitometric analysis of blots (n = 4) with specific p-Erk1/2 levels normalized to the corresponding tubulin levels. Bars represent the mean ±S.E.M. ** $p<0.01$ vs untreated cells. (C) Densitometric analysis of blots (n = 4) with specific p-Akt levels normalized to the corresponding tubulin levels. Bars represent the mean ±S.E.M. ** $p<0.01$ vs untreated cells. (D) Hek-D3R cells were treated with Quin (1 μM; 5 min) in the absence or in the presence of both LY294002 (LY; 10 μM) and H89 (1 μM), added 30 min before agonist stimulation and analysed for p-Erk1/2 and p-Akt by WB; a representative WB is shown. (E) Densitometric analysis of blots (n = 4) with specific p-Erk1/2 levels normalized to the corresponding tubulin levels. Bars represent the mean ±S.E.M. *** $p<0.01$ vs

untreated cells; ** $p<0.01$ vs untreated cells. (F) Densitometric analysis of blots (n = 4) with specific p-Akt levels normalized to the corresponding tubulin levels. Bars represent the mean ±S.E.M. *** $p<0.01$ vs untreated cells. (G) Hek-D3R cells were incubated overnight with PTX (100 ng/ml) following by Quin incubation (1 μM) for 5 min and analysed for p-Erk1/2 and p-Akt by WB; a representative WB is shown. (H) Densitometric analysis of blots (n = 4) with specific p-Erk1/2 levels normalized to the corresponding tubulin levels. Bars represent the mean ±S.E.M. *** $p<0.01$ vs untreated cells. (I) Densitometric analysis of blots (n = 4) with specific p-Akt levels normalized to the corresponding tubulin levels. Bars represent the mean ±S.E.M. *** $p<0.01$ vs untreated cells. (J) Hek-D3R cells were treated with Quin (1 μM; 5 min) in the absence or in the presence of LY294002 (LY; 10 μM) added 30 min before agonist stimulation and analysed for p-Erk1/2 and p-Akt by WB; a representative WB is shown. (K) Densitometric analysis of blots (n = 4) with specific p-Erk1/2 levels normalized to the corresponding tubulin levels. Bars represent the mean ±S.E.M. *** $p<0.01$ vs untreated cells (L) Densitometric analysis of blots (n = 4) with specific p-Akt levels normalized to the corresponding tubulin levels. Bars represent the mean ±S.E.M. *** $p<0.001$ vs untreated cells. Data were statistically analyzed by one-way ANOVA followed by post-hoc comparison with Bonferroni test.

S3. In Hek-D1R/D3R cells either D1R or D3R antagonists prevent the D1R/D3R heterodimer-mediated activation of Erk1/2.

To evaluate the impact of either D1R or D3R antagonists on D1R/D3R heterodimer activation of Erk1/2, Hek-D1R/D3R cells were incubated for 30 min with either the D1R antagonist, SCH23390 (10 μM), or with the D2R/D3R antagonist, sulpiride (10 μM), followed by the D1R agonist SKF81297 (1 μM; 5 min) or the D2R/D3R agonist quinpirole (1 μM; 5 min) and analyzed for p-Erk1/2. In preliminary experiments, SCH2339 and sulpiride at 10 μM was the most effective concentration able to prevent both D1R- and D3R-induced activation of Erk1/2, respectively (data not shown). As previously demonstrated [18], we found that activation of Erk1/2 by SKF81297 was specifically prevented by pre-incubating cells with either SCH23390 or with sulpiride (Figure S4A and B). Similarly, Erk1/2 activation induced by quinpirole was significantly reduced by blocking either D1R or D3R by SCH23390 (1 μM) or sulpiride (1 μM), respectively (Figure S4C and D). In addition, Hek-D1R/D3R cells were incubated for 30 minutes with either the SCH23390 or sulpiride followed by DA (1 μM) for 5 minutes. As shown in Figure S4E and F, both antagonists prevented the activation of Erk1/2 induced by DA.

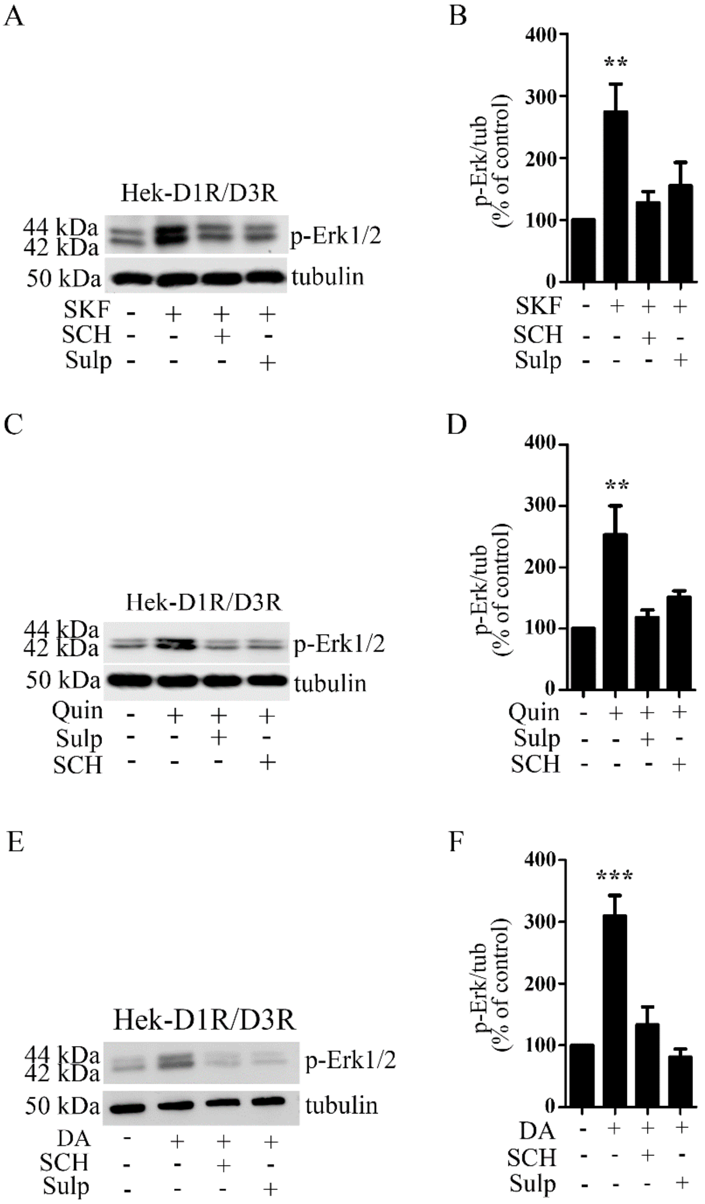

Figure S3 Reduced activation of Erk1/2 pathway by either D1R or D3R antagonists in Hek D1R/D3R cells. (A) Hek D1R/D3R cells were exposed to SKF (1 μM; 5 min) in the presence or in absence of the D1R antagonist SCH23390 (SCH, 10 μM) or the D3R antagonist sulpiride (Sulp, 10 μM) added 30 min before, and analyzed for p-Erk1/2 by WB; a representative WB is shown. (B) Densitometric analysis of blots (n=4) with specific p-Erk1/2 signals normalized to the corresponding tubulin levels. Bars represent the mean ±S.E.M. ***p<0,001 vs untreated cells. (C) Hek D1R/D3R cells were exposed to the Quin (1 μM; 5 min) in the presence or in absence of SCH (10 μM) or Sulp (10 μM) added 30 min before and analyzed for p-Erk1/2 by WB; a representative WB is shown. (D) Densitometric analysis of blots (n=4) with specific signals of p-Erk1/2 normalized to the corresponding tubulin levels. Bars represent the mean ±S.E.M. ***p<0,001 vs untreated cells. (E) Hek D1R/D3R cells were exposed to DA (1 μM; 5 min) in the presence or in absence of SCH (10 μM) or Sulp (10 μM) added 30 min before stimulation, and analyzed for p-Erk1/2; a representative WB is shown. (F) Densitometric analysis of blots (n=4) with specific p-Erk1/2 levels normalized to the corresponding tubulin levels. Bars represent the mean ±S.E.M. ***p<0,001 vs untreated cells. Data were statistically analyzed by one-way ANOVA followed by post-hoc comparison with Bonferroni test.

S4. Silencing of beta arrestin-1 and beta arrestin-2 in primary mouse striatal neurons by siRNA technique

To investigate the role of beta-arrestin 1 and 2 in the D1R/D3R heterodimer-induced activation of Erk1/2 in primary cultures of mouse striatal neurons, the siRNA approach was used. First, striatal neurons were individually treated with two different siRNA sequences for both beta-arrestin 1 (si-bArr1a and si-bArr1b)

and beta-arrestin 2 (si-bArr2a and si-bArr2b) at 25-50nM for 120 h and analysed for beta-arrestin 1 and 2 expression levels by WB (Figure S5A-D). si-bArr1b and si-bArr2a were selected as the most efficient and specific siRNAs and used for the subsequent experiments. Striatal neurons were then treated with either si-bArr1b or si-bArr2a at two different concentrations (25 nM–50 nM) for 120h, showing that the 50nM was the most efficient concentration in silencing striatal beta arrestin-1 and beta-arrestin-2, respectively (Figure S5E and F and Figure S5G and H); the selectivity of si-bArr1b (50nM; 120h) in silencing striatal beta arrestin-1 but not beta-arrestin 2 and of si-bArr2a in silencing striatal beta-arrestin-2, but not beta-arrestin-1, was also demonstrated (Figure S5I-K).

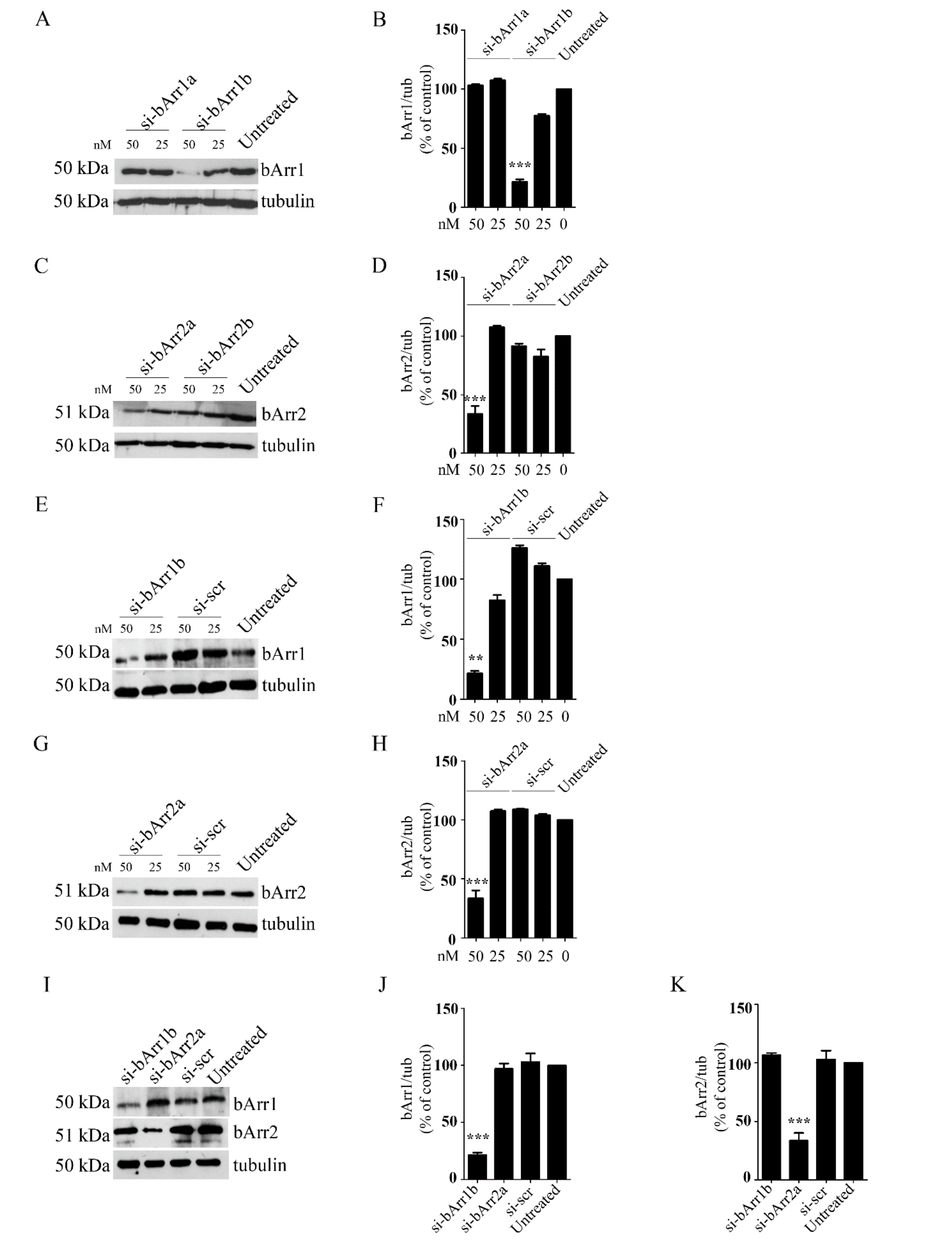

Figure S4. Knockdown of beta-arrestin1 and beta-arrestin2 expression by siRNA. A) Striatal neurons were treated with si-bArr1a and si-abArr1b (25 and 50nM) for 120 h and analysed for beta-arrestin1 (bArr1); a representative WB blot is shown. B) Densitometric analysis of blots (n = 3) with specific bArr1 levels normalized to the corresponding tubulin levels. Bars represent the mean ±S.E.M. *** p<0.001 vs untreated cells. C) Striatal neurons were treated with si-bArr2a and si-abArr2b (25 and 50nM) for 120 h and analysed for beta-arrestin2 (bArr2); a representative WB blot is shown. D)

Densitometric analysis of blots (n = 3) with specific bArr2 levels normalized to the corresponding tubulin levels. Bars represent the mean ±S.E.M. *** p<0.001 vs untreated cells. E) Striatal neurons were treated with si-bArr1b and si-scr (25 and 50nM) for 120 h and analysed for bArr1; a representative WB blot is shown. F) Densitometric analysis of blots (n = 3) with specific bArr1 levels normalized to the corresponding tubulin levels. Bars represent the mean ±S.E.M. ** p<0.01 vs untreated cells. G) Striatal neurons were treated with si-bArr2a and si-scr (25 and 50nM) for 120 h and analysed for beta-bArr2; a representative WB blot is shown. H) Densitometric analysis of blots (n = 3) with specific bArr2 levels normalized to the corresponding tubulin levels. Bars represent the mean ±S.E.M. *** p<0.001 vs untreated cells. I) Striatal neurons were treated si-bArr1b or si-abArr2a or si-scr (50nM) for 120 h and analysed for bArr1 and bArr2; a representative WB blot is shown. J-K) Densitometric analysis of blots (n = 3) with specific bArr1 and bArr2 levels normalized to the corresponding tubulin levels. Bars represent the mean ±S.E.M. *** p<0.001 vs untreated cells.
